# Influence of two anti-tumor drugs, pazopanib, and axitinib, on the development and thyroid-axis of zebrafish (*Danio rerio*) embryos/larvae

**DOI:** 10.3389/fendo.2023.1204678

**Published:** 2023-07-31

**Authors:** Liu Yang, Ping-hui Tu, Cao-xu Zhang, Rong-rong Xie, Mei Dong, Yu Jing, Xia Chen, Gang Wei, Huai-dong Song

**Affiliations:** ^1^ Department of Molecular Diagnostics, The Core Laboratory in Medical Center of Clinical Research, Shanghai Jiaotong University School of Medicine, Shanghai, China; ^2^ Department of Endocrinology, Shanghai Ninth People’s Hospital, State Key Laboratory of Medical Genomics, Shanghai Jiaotong University School of Medicine, Shanghai, China; ^3^ Key Laboratory of Environmental Pollution Monitoring and Disease Control, Ministry of Education, Guizhou Medical University, Guiyang, China; ^4^ Beijing Key Laboratory of Diabetes Research and Care, Department of Endocrinology, Beijing Diabetes Institute, Beijing Tongren Hospital, Capital Medical University, Beijing, China; ^5^ Department of Endocrinology, Shanghai Gongli Hospital, Shanghai, China; ^6^ Department of Endocrinology and Metabolism, Shanghai Fourth People’s Hospital Affiliated to Tongji University School of Medicine, Shanghai, China

**Keywords:** thyroid disruption, hypothyroidism, thyroid histomorphology, hypothalamuspituitary-thyroid (HPT) axis, zebrafish

## Abstract

**Introduction:**

In recent years, the potential toxicities of different pharmaceuticals toward the thyroid system have received increasing attention. In this study, we aim to evaluate the toxic effects of pazopanib and axitinib, two anti-tumor drugs with widespread clinical use, on thyroid function in the zebrafish model.

**Methods:**

We measured levels of thyroid-related hormones using the commercial Enzyme-Linked Immunosorbent Assay (ELISA) kit. Whole-mount in situ hybridization (WISH) analysis was employed to detect target gene expression changes. Morphology of the thyroid were evaluated by using transgenic Tg (*tg*: EGFP) fish line under a confocal microscope. The relative mRNA expression of key genes was verified through quantitative real-time polymerase chain reaction (RT‒qPCR). The size and number of the follicles was quantified whereby Hematoxylin–Eosin (H & E) staining under a light microscope.

**Results:**

The results revealed that fertilized zebrafish embryos were incubated in pazopanib or axitinib for 96 hours, development and survival were significantly affected, which was accompanied by significant disturbances in thyroid endocrine system (e.g., increased thyroid-stimulating hormone (TSH) content and decreased triiodothyronine (T3) and thyroxine (T4) content, as well as transcription changes of genes associated with the hypothalamus-pituitary-thyroid (HPT) axis. Moreover, based on whole-mount in situ hybridization staining of tg and histopathological examination of zebrafish embryos treated with pazopanib and axitinib, we observed a significantly abnormal development of thyroid follicles in the Tg (*tg*: EGFP) zebrafish transgenic line.

**Conclusion:**

Collectively, these findings indicate that pazopanib and axitinib may have toxic effects on thyroid development and function, at least partially, by influencing the regulation of the HPT axis. Thus, we believe that the potential thyroid toxicities of pazopanib and axitinib in their clinical applications should receive greater attention.

## 1 Introduction

Recently, there has been increasing concern regarding the occurrence, fate, and toxic effects of pharmaceutical residues in the aquatic environment (1–5). To date, wastewater treatment plants have rarely been explicitly designed to filter drugs, and thus residual amounts have been discharged directly into the environment without any adequate restrictions (6). This has led to the continuous entry and accumulation of pharmacologically active substances in the aquatic environment. Notably, pharmaceuticals, especially molecular targeted drugs, may have toxic effects on thyroid axis of aquatic organisms, posing a major threat to public health (7, 8).

Tyrosine kinase inhibitors (TKIs), a new class of molecular multi-targeted anticancer drugs, have recently been used to treat neuroendocrine neoplasia (NEN), aggressive gastrointestinal stromal tumor (GIST), hepatocellular carcinoma (HCC), renal cell carcinoma (RCC), and thyroid cancer (9–11). Although these agents are generally recognized as less toxic than traditional cytotoxic chemotherapy, certain side effects are still evident, including hypertension and fatigue. However, the most common adverse effect is thyroid dysfunction, such as hypothyroidism (12). For example, in China, the use of two types of TRI, pazopanib and axitinib, which have been approved for the first- and second-line therapy of RCC, has been associated with a higher incidence of hypothyroidism of approximately 3% to 18% and 12% to 89%, respectively (12–18). Consistently, several studies have reported that patients with metastatic renal cancer treated with pazopanib and axitinib often have abnormal thyroid function, as indicated by elevated serum thyroid-stimulating hormone (TSH) levels (13, 19). Numerous studies have also reported a higher frequency of thyroid disorders in patients taking pazopanib and axitinib (15–17, 20); however, neither the associated molecular mechanisms nor the clinical features of these adverse effects have been established.

Within the endocrine system, the thyroid gland plays a vital role in the homeostatic control of fundamental physiological processes in vertebrates, including body growth and energy expenditure (21), and within this system, the hypothalamic–pituitary–thyroid (HPT) axis controls normal thyroid function by regulating the synthesis and metabolism of thyroid hormones (THs), which also maintains TH homeostasis *via* a negative feedback loop.

In this study, we aimed to assess the developmental toxicities of pazopanib and axitinib in a clinical setting and further demonstrate the potential mechanisms underlying the development of hypothyroidism induced by TKIs using the zebrafish model. Ecotoxicological data are scarce for TKIs currently in use (22). So, we chose a conservative concentration in the reference with clinical dose during the experiments. The findings of this study will provide valuable evidence for the hormonal and organismic effects of pazopanib and axitinib, particularly regarding thyroid disruption.

## 2 Materials and methods

### 2.1 Chemicals

Pazopanib (Cat. no. HY-10208) and axitinib (Cat. no. H-10065) were obtained from MedChemExpress (China). During the experiments, stock solutions of pazopanib and axitinib were prepared by dissolving in DMSO at 10 mM and stored at -20°C.

### 2.2 Zebrafish husbandry and chemical treatment

Tg (*tg*: EGFP) was purchased from the China Zebrafish Resource Center. Zebrafish embryos were raised at 28.5°C. All methods were performed according to the approved guidelines of Shanghai Jiao Tong University School of Medicine. For chemical treatment, fertilized embryos (~2 hpf) were randomly divided into 12-well plates for 96 hours, containing 1 mL of pazopanib and axitinib exposure solutions at different concentrations (10, 50, and 100 nM). Control embryos were incubated with the same concentration of DMSO.

### 2.3 Hormone extraction and measurement

Thyroid-related hormones [triiodothyronine (T3), thyroxine (T4), and TSH] in zebrafish embryos were extracted according to the manufacturer’s instructions (CAMILO, China, cat. no. 2Z-KMLJ784005 for T3, cat. no. 2Z-KMLJ784024 for T4, and cat. no. 2Z-KMLJ784017 for TSH). briefly, 600 embryos each group were collected at 96 hpf, homogenized in Phosphate-buffered saline (PBS buffer) in a clean 1.5 mL tube, and finally sonicated (Sonics and Materials Vibra-Cell at 50% output for 20 seconds). Homogenized samples were centrifuged at 13 000 rpm for 30 minutes at 4°C, and transferred the supernatant to another 1.5 mL tube. The hormone levels were measured with a microplate reader (Bio Tek, USA), and the results of each treatment were normalized to those of the control group.

### 2.4 WISH evaluation

Anti-sense RNA probes were transcribed by using a Digoxigenin (DIG) -RNA labelling kit and purified with Quick Spin Columns (Roche). All primers used for RNA probe transcription was listed in **Table S1**.

WISH analysis was performed as previously described (23, 24). Briefly, Zebrafish embryos at 96 hpf were collected and fixed overnight in 4% paraformaldehyde at 4°C. Then embryos were dehydrated in 25%, 50%, and 75% methanol consequently and then stored in 100% methanol solution at -20°C overnight. Dehydrated embryos were rehydrated in 75%, 50%, and 25% methanol consequently, and then treated with proteinase K (100 µg/mL) at room temperature (RT) for 30 minutes, riboprobe (0.5~1 ng/µL) hybridization was performed at 68°C overnight. After hybridization, embryos were rinsed through a graded series of standard saline citrate (2× SSC containing 50% formamide, 2x SSC, 0.2x SSC) (Sigma) for 30 minutes. Simples were then incubated overnight with anti-digoxigenin-AP Fab fragments antibody (Roche) at 4°C overnight. Finally, NBT/BCIP color reaction kit (Vector Laboratories, SK-5400) was used for WISH stain and pictures were taken under an SMZ25 dissecting microscope (Nikon). The indicated areas for thyroid and pituitary were quantified by the Image J software.

### 2.5 Confocal microscopy evaluation

Zebrafish embryos were anesthetized (tricaine, 0.016%), and were then embedded in dishes containing 1.2% low-melting agarose (Sangon, China). A Nikon A1 confocal laser microscope was used to capture confocal images. Analyses of the fluorescence images were performed using Imaris and Image J software.

### 2.6 RNA isolation and quantitative real-time PCR

Total RNA was extracted from zebrafish using TRIzol reagent (Invitrogen, USA). After treatment with gDNA Eraser at 42°C for 2 mins, 1 μg of total RNA was reverse transcribed using random hexamers and oligo dT primers according to the manufacturer’s instructions (Takara, RR047A). The TB Green^®^ Premix Ex Taq™ (Tli RNaseH Plus) (Takara, RR420A) was used for the qPCR analysis on the QuantStudio 6 Flex Real-Time PCR System (ABI). qPCR program sets as follow: Initial denaturation at 95°C for 30 s (step 1), anneal primers and extend the DNA for 34 seconds at 60°C after denaturation at 95°C for 5 s (step 2, 40 cycles). The relative expression values were normalized against the internal control β-actin (actb1) gene. Sequences of the PCR primers were listed in **Supplementary Table S2**.

### 2.7 Hematoxylin–Eosin staining

Zebrafish embryos were fixed in 4% PFA and then embedded in paraffin. The zebrafish embryos were sectioned (5 μm) and stained with H&E. Section images were captured using Nikon Eclipse Ni-U Microscope (Nikon, Japan) under a light microscope.

### 2.8 Statistical analysis

All data are expressed as mean ± SD and were performed using unpaired Student’s t-test or one-way analysis of variance (ANOVA). GraphPad Prism 8 software and Excel were used for statistical analyses. Values of *P <*0.05 were statistically significant.

## 3 Results

### 3.1 Developmental toxicity and survival of zebrafish embryos

**Figure 1:**
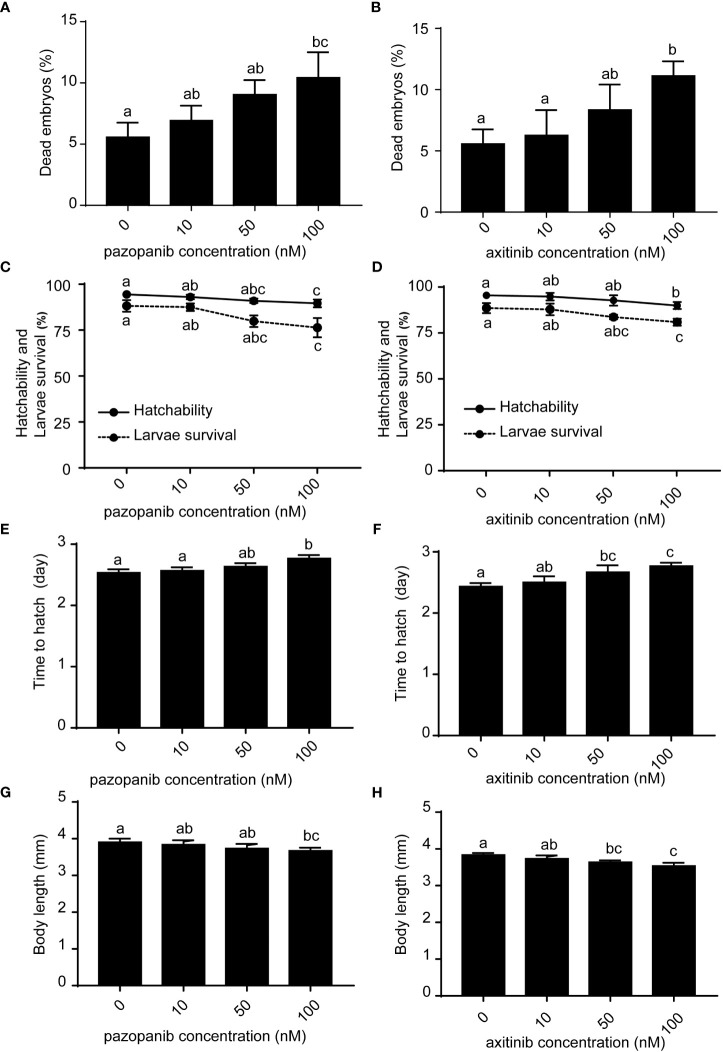
Developmental toxicities and survival of zebrafish embryos following exposure to different concentrations (0, 10, 50, 100 nM) of pazopanib and axitinib for 96 (h) **(A, B)** Dead embryos, **(C, D)** hatchability and larval survival rates, **(E, F)** time to hatching (days), and **(G, H)** body length (mm). Relative thyroid follicle numbers were analyzed from D-F. Data are expressed as the mean ± SD, n = 3 (48 embryos per concentration, with three replicates). Different letters indicate a statistically significant difference between groups, *P* < 0.05, One-way ANOVA test followed by Tukey’s multiple comparison.

Zebrafish embryos (~2 hpf) were randomly selected and exposed to pazopanib or axitinib at four different doses [0 (control), 10, 50, and 100 nM] for 96 h. Our results showed that compared with the control groups, dead embryos increased at 6 hpf following exposure to pazopanib and axitinib (**Figures 1A, B**), which led to further marked reductions in embryo survival and hatchability (**Figures 1C, D**). Furthermore, relative to the control group, we recorded a significant delay in the hatching time of zebrafish embryos exposed to pazopanib and axitinib (**Figures 1E, F**) and found that the body lengths of embryos exposed to pazopanib and axitinib were shorter than those of the control group (**Figures 1G, H**). Notably, the developmental toxicity of pazopanib and axitinib in zebrafish embryos increased gradually with increasing drug concentration, with the latter being established to be more toxic than the former (**Figure 1**).

### 3.2 Dysregulated thyroid-related hormones in zebrafish embryos

**Figure 2:**
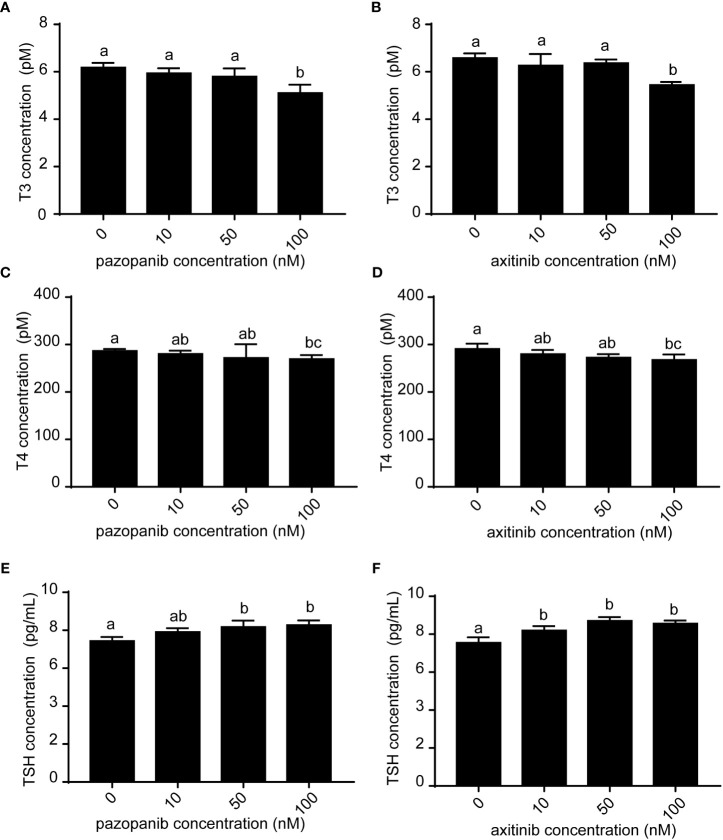
Levels of T3, T4, and TSH in zebrafish embryos following exposure to different concentrations (0, 10, 50, 100 nM) of pazopanib and axitinib for 96 h. **(A, C, E)** represent pazopanib toxicity, and **(B, D, F)** represent axitinib toxicity. Relative thyroid follicle numbers were analyzed from **(D-F)**. Data are expressed as the mean ± SD, n = 3 (20 embryos per concentration, with three replicates). Different letters indicate a statistically significant difference between groups, *P* < 0.05, One-way ANOVA test followed by Tukey’s multiple comparison.

We subsequently evaluated the thyroid disruption in zebrafish embryos following exposure to pazopanib and axitinib for 96 h (**Figure 2**). Whole-body levels of T3 and T4 in the exposure groups were found to be significantly lower than those in the control group (**Figures 2A-D**). In contrast, we observed a marked elevation in the concentration of TSH in zebrafish embryos in response to exposure to pazopanib and axitinib (**Figures 2E, F**). These results indicate that pazopanib and axitinib can induce an appreciable dysregulation of thyroid-related hormones in zebrafish embryos, thereby indicating disordered thyroid hormone function.

### 3.3 A negative feedback regulation due to defective thyroid function in zebrafish embryos

**Figure 3:**
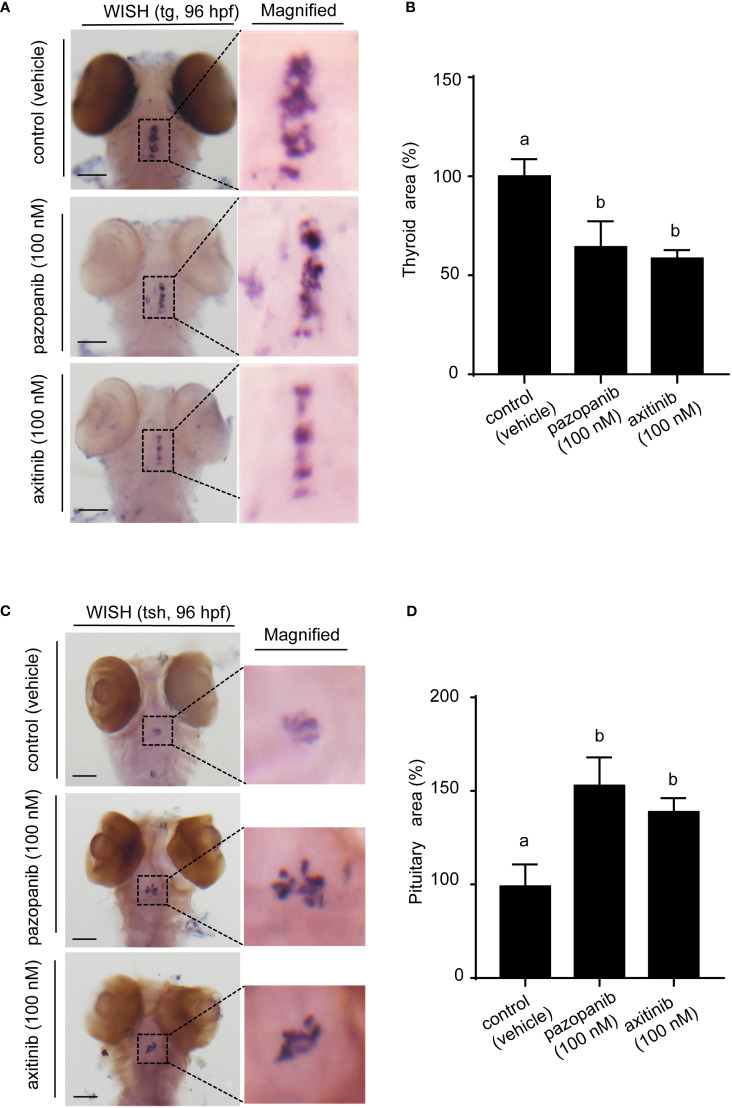
Whole-mount *in situ* hybridization (WISH) of *tg* and *tshβ* in zebrafish embryos following exposure to 100 nM of pazopanib and axitinib for 96 h **(A)** WISH of *tg*. **(B)** The relative reduction in thyroid area (%). **(C)** WISH of *tshβ*. **(D)** Relative reduction in thyroid area (%). Data are expressed as the mean ± SD, n = 3 (20 embryos per concentration, with three replicates). Different letters indicate a statistically significant difference between groups, *P* < 0.05, One-way ANOVA test followed by Tukey’s multiple comparison.

Given the observed changes in thyroid-related hormones in response to drug exposure, we used WISH staining of *tg* to verify the toxic effects of pazopanib or axitinib on the thyroid gland and its associated function in TH synthesis and secretion. Our staining results revealed that compared with those exposed to the vehicle, there was a noticeable alteration in thyroid morphogenesis (e.g., hypotrophy of thyroid follicles) in zebrafish embryos following exposure to pazopanib or axitinib for 96 h, which is taken to be indicative of marked thyroid hypoplasia (**Figure 3A**). In particular, relative to the control group, we detected a striking reduction in the size of the thyroid in zebrafish embryos exposed to pazopanib and axitinib (-64.37% and -58.51%, respectively) (**Figures 3A, B**), which is consistent with the results indicating defective thyroid function in response to pazopanib and axitinib exposure.

Given the increased TSH content in zebrafish embryos following exposure, we also used WISH staining of *tsh* to evaluate pathological changes in the pituitary gland. Interestingly, we noted that treatment with both pazopanib and axitinib resulted in a large and diffuse pituitary phenotype, with an increased pituitary area and uneven distribution (**Figures 3C, D**). Collectively, these results, taken in conjunction with the increase in TSH content, indicate that exposure to pazopanib and axitinib triggers a putative negative feedback loop that seeks to restore normal thyroid function in zebrafish embryos.

### 3.4 Abnormal thyroid development and morphogenesis in zebrafish embryos

**Figure 4:**
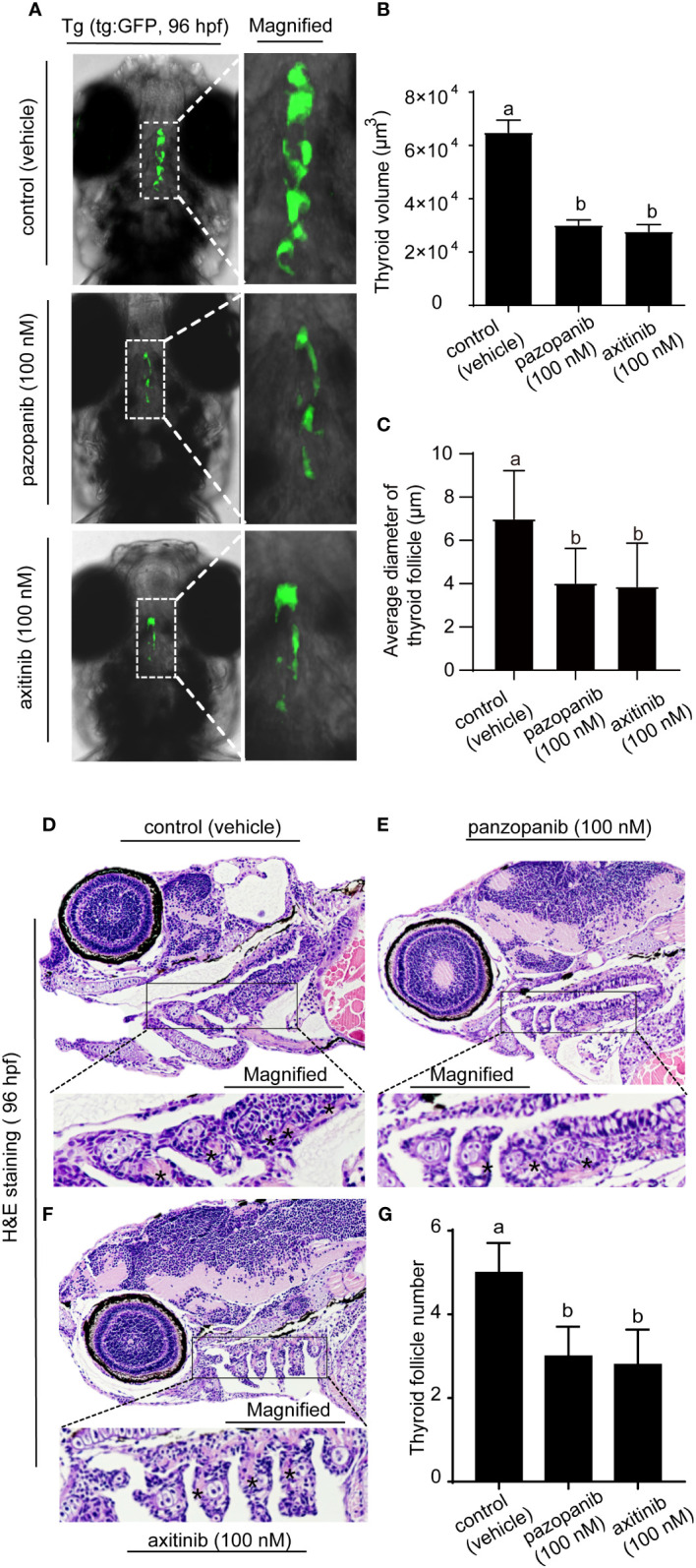
Morphology and histopathology of the thyroid in zebrafish embryos following exposure to 100 nM of pazopanib and axitinib for 96 h **(A)** Confocal imaging analyses of the thyroid in transgenic Tg (*tg:* EGFP) embryos. **(B)** The relative reduction in thyroid volume (%) from **(A, D-F)** H&E staining of zebrafish embryos. **(C)** The size of the follicles was analyzed from D-F. **(G)** Relative thyroid follicle numbers were analyzed from D-F. Data are expressed as the mean ± SD, n = 6. Different letters indicate a statistically significant difference between groups, *P* < 0.05, One-way ANOVA test followed by Tukey’s multiple comparison.

We subsequently validated the marked pathological changes in zebrafish embryos exposed to pazopanib or axitinib for 96 h by labeling thyroid follicular cells using the Tg (*tg*:EGFP) zebrafish transgenic line (**Figures 4A, B**), performed in conjunction with histological and morphometric analyses (**Figures 4C-E**). Confocal live imaging revealed that exposure to pazopanib and axitinib promoted a pronounced retardation of thyroid gland development in zebrafish embryos, which in turn led to a marked reduction (100% vs. 53.60%, 100% vs. 57.30%, respectively) in the thyroid volume (**Figures 4A, B**, **Supplementary movie S1-S3**.)

Moreover, histological analysis revealed that exposure to pazopanib and axitinib was associated with the development of a smaller follicle colloid lumen, accompanied by hypotrophy of the follicular epithelium, which can probably be attributed to the hypoplasia of follicle cells (**Figures 4D-F**). Consistently, compared with the control groups, we detected a marked reduction in the size of the follicles (**Figure 4C**) and the number of thyroid follicles following exposure to pazopanib and axitinib (5 vs. 3.1, 5 vs. 2.8, respectively) (**Figure 4G**).

### 3.5 Transcriptional changes in zebrafish genes associated with thyroid development and function

**Figure 5:**
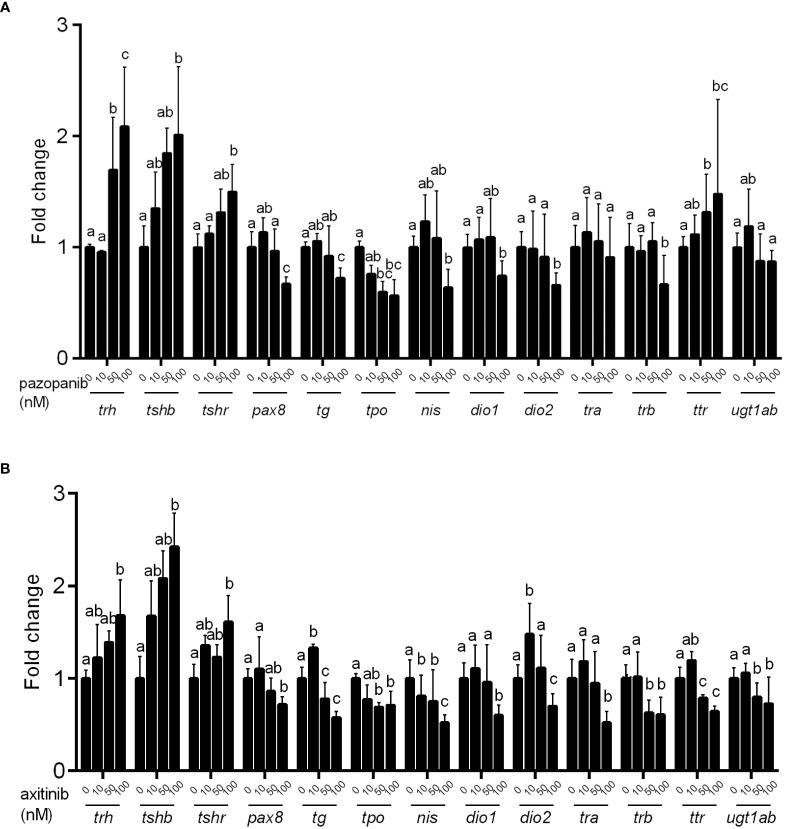
Relative expression of hypothalamic–pituitary–thyroid (HPT) axis related genes in zebrafish embryos following exposure to different concentrations (0, 10, 50, 100 nM) of pazopanib and axitinib for 96 h. **(A, B)** for pazopanib and axitinib, respectively. Data are expressed as the mean ± SD, n = 6 (60 embryos per concentration, with three replicates). Different letters indicate a statistically significant difference between groups, *P* < 0.05, One-way ANOVA test followed by Tukey’s multiple comparison.

Using qPCR, we systematically examined alterations in the transcriptional levels of key genes along the HPT axis. As shown in **Figure 5**, the exposure of zebrafish embryos to pazopanib or axitinib for 96 h induced notable alterations in the mRNA levels of genes associated with thyroid development and function. In particular, we detected significant increases in the transcription levels of *trh*, *tshβ*, and *tshr* in the pazopanib- or axitinib-treated groups, whereas marked reductions were detected in the expression of *pax8*, *tg*, *tpo*, *nis*, *dio1*, *dio2*, *trβ*, and *ugt1ab* (**Figures 5A, B**). Notably, whereas we observed reductions in the mRNA expression of *trα* and *ttr* in the axitinib-treated group (**Figure 5B**), no significant changes were observed for *trα* in the pazopanib-treated group, whereas the levels of *ttr* were markedly induced (**Figure 5A**).

## 4 Discussion

Cancer is a major public health problem worldwide, and its incidence is increasing annually (25). However, if a patient’s metabolic waste is not properly managed, the excretion of residual drugs becomes latent and poses a risk of environmental pollution. Pazopanib and axitinib are selective multitargeted receptor TKIs that exhibit potent activity against many tumors, especially clear cell renal carcinomas (26, 27). Although they have validated efficacy and safety (13, 19), thyroid dysfunction is a well-known adverse effect caused by them (12).

In this study, we analyzed the development and survival of zebrafish embryos exposed to different concentrations of pazopanib and axitinib at 96 hpf for the first time. We found that exposure to pazopanib and axitinib promoted significant developmental toxicity and increased mortality among zebrafish embryos. In particular, exposure to pazopanib and axitinib induced a longer hatching time, leading to shorter body lengths in zebrafish embryos. By determining the expression of thyroid-related hormones, we observed that pazopanib- and axitinib- treated embryos exhibited notable reductions in T3 and T4 levels, accompanied by a hypothyroidic phenotype. Collectively, these results indicated that exposure to pazopanib and axitinib can lead to thyroid endocrine disruption in zebrafish embryos, causing further developmental retardation.

To precisely evaluate the toxic effects of pazopanib or axitinib on the thyroid gland, we conducted WISH staining (using anti-sense *tg* RNA probes) in zebrafish embryos. Interestingly, we noted an obvious arrested in the development of thyroid morphogenesis in response to pazopanib or axitinib, as evidenced by the hypotrophy of thyroid follicles and reduction in thyroid size, thereby indicating a deficit in thyroid function. Given that exposure to both pazopanib and axitinib can induce significant thyroid disruption and promote the elevation of TSH levels in zebrafish embryos, we also conducted WISH staining of *tsh* to evaluate pathological changes in the pituitary gland. Consistently, we observed a large and diffuse pituitary phenotype (i.e., an enlarged pituitary area and uneven distribution) following exposure to pazopanib or axitinib, clearly reflecting the expansion of the pituitary gland. Collectively, these results, together with the evaluated TSH levels, demonstrated that exposure to pazopanib or axitinib could cause marked thyroid disruption, triggering a negative feedback loop to restore thyroid function in zebrafish embryos.

In this study, we also used Tg (*tg:* EGFP) transgenic zebrafish embryos for dynamic phenotypic analysis of thyroid morphogenesis. Consistent with the WISH staining results, we verified that confocal live imaging revealed that pazopanib and axitinib can induce a striking retardation in thyroid development in transgenic zebrafish embryos. Furthermore, we performed a histological analysis of thyroid gland tissues to determine whether exposure to pazopanib and axitinib can affect thyroid structure and function and establish the underlying mechanisms. In line with expectations, we found that exposure to both pazopanib and axitinib resulted in significant thyroid toxicity, manifested in changes in thyroid histology, including a smaller follicular colloid cavity and follicular epithelial cell dysplasia. Pax8 is a specific transcription factor in the thyroid gland and contributes to the maturation of follicular cells. Interestingly, qPCR analysis showed that the mRNA expression levels of pax8 declined markedly in response to pazopanib and axitinib exposure, further demonstrating that these two compounds can cause defective thyroid development.

We utilized quantitative qPCR to investigate the transcriptional changes of major genes associated with the HPT axis. Accordingly, we established that exposure to pazopanib and axitinib induced marked increases in the expression of genes related to the synthesis and release of THs, which provides further evidence in support of the implicated negative feedback mechanism (i.e., compensation for the reduced levels of THs) at the molecular level. We also found that exposure to pazopanib and axitinib resulted in an obvious reduction in the expression of genes associated with the binding, transport, and action of TH, which may contribute to the impairment of thyroid function. Moreover, in response to exposure to pazopanib and axitinib, we detected marked reductions in the mRNA expression levels of genes associated with TH metabolism in zebrafish embryos, providing further evidence of dysregulated thyroid function (28, 29). Collectively, the presence of TH in the serum reflects the immediate effects of pazopanib and axitinib exposure on the HPT axis, whereas the histomorphology of the thyroid serves as an indicator of a more persistent and integrated toxic effect associated with TH physiology (30).

Recently, environmental pollution-related factors, such as drugs that interfere with thyroid function, have been linked to an increased incidence of hypothyroidism (31, 32). However, there are no guidelines regarding the frequency of thyroid function test monitoring or treating thyroid dysfunction induced by drugs such as TKIs. The results of this study not only provide valuable insights into the thyroid toxicity of pazopanib and axitinib but can also contribute to developing higher standard screening programs for specific high-risk categories (e.g., newborns exposed to particular drugs) by analyzing the alterations in gene expression along the HPT axis as well as changes in thyroid histomorphology.

**Figure 6:**
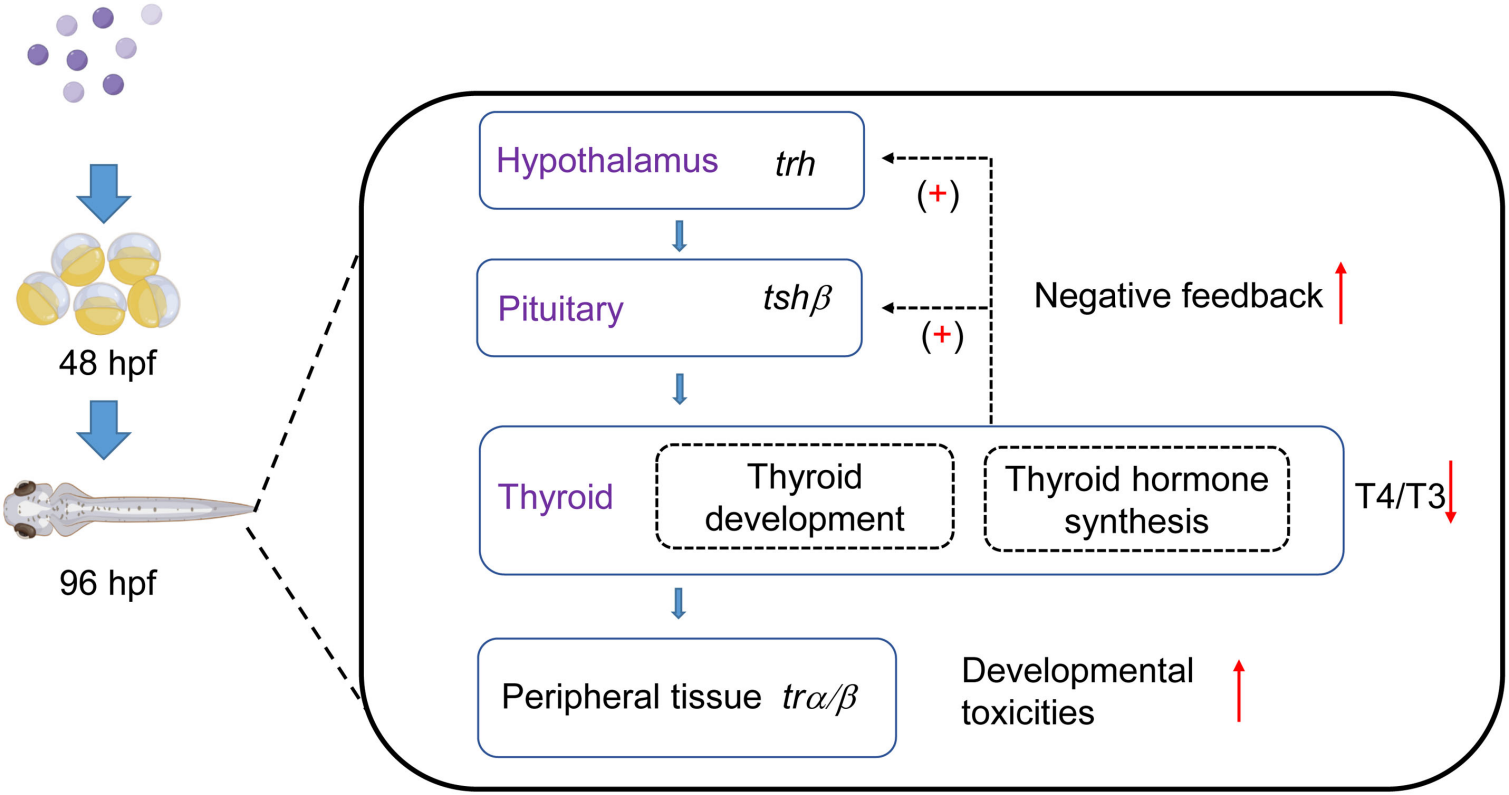
Influence of two tyrosine kinase inhibitors on development and the thyroid system in zebrafish embryos. Pazopanib and axitinib were found to have notable toxic effects on the development and survival of zebrafish embryos, which is closely associated with disordered thyroid function. This figure was modified using Figdraw and PowerPoint.

In this study, we considered that the TKIs, pazopanib and axitinib, could promote significant toxic effects on the development and survival of zebrafish embryos, which were closely associated with disordered thyroid function (**Figure 6**). Moreover, exposure to zebrafish embryos induces a marked disturbance in the homeostasis of the thyroid hormone system, which is, at least partially, attributable to regulation of the HPT axis and destruction of the thyroid follicle structure. Additionally, the results of this study provide new evidence for thyroid toxicity caused by targeted therapy, which indicates that TKIs such as pazopanib and axitinib should be scientifically evaluated in clinical applications.

We evaluated the adverse effects of pazopanib and axitinib on the developmental toxicities and thyroid endocrine disruption by using zebrafish model. In zebrafish, the thyroid endocrine system shows conserved mechanisms as compared with humans (33). Thyroid hormone (TH) for T4 production in thyroid follicle begins about 72hpf (34). Moreover, numerous reports have proved that the toxicity profiles of various chemical compounds and drugs in zebrafish are strikingly consistent with mammalian models (35). However, we did not continue the related study in mice, and we will further reveal the mechanism of TKI-induced thyroid dysfunction in mice.

## Data availability statement

The original contributions presented in the study are included in the article/**Supplementary Material**. Further inquiries can be directed to the corresponding authors.

## Ethics statement

The animal study was reviewed and approved by The Ethics Committee of Shanghai Ninth People’s Hospital affiliated with the Shanghai Jiao Tong University School of Medicine.

## Author contributions

LY and GW: Conceptualization, Writing the original draft. P-HT, R-RX, YJ: Methodology, Investigation, Validation, Formal analysis. GW: Supervision, Writing-Review and Editing. LY, C-XZ, and H-DS: Resources, Visualization. MD and XC: Funding acquisition. All authors contributed to the article and approved the submitted version.
