# Supplementary material for: Influence of two anti-tumor drugs, pazopanib, and axitinib, on the development and thyroid-axis of zebrafish (Danio rerio) embryos/larvae
Source: Front Endocrinol (Lausanne). 2023 Jul 31;14:1204678. doi: 10.3389/fendo.2023.1204678 (PMC10433177; doi:10.3389/fendo.2023.1204678)
Supplement: Supplementary file 1 [file Table_1.docx]

Influence of two anti-tumor drugs, pazopanib, and axitinib, on the development and thyroid-axis of zebrafish (*Danio rerio*) embryos/larvae

**Liu Yang^1^, Ping-hui Tu^1^, Cao-xu Zhang^1,2^, Rong-rong Xie^3^, Mei Dong^1^, Yu Jing^1^, Xia Chen^4^, Gang Wei^1,2,3,5^*, Huai-dong Song^1^*******

^1^ Department of Molecular Diagnostics, The Core Laboratory in Medical Center of Clinical Research, Department of Endocrinology, Shanghai Ninth People’s Hospital, State Key Laboratory of Medical Genomics, Shanghai Jiaotong University School of Medicine, Shanghai 200011, China

^2^ Key Laboratory of Environmental Pollution Monitoring and Disease Control, Ministry of Education, Guizhou Medical University

^3^ Beijing Key Laboratory of Diabetes Research and Care, Department of Endocrinology, Beijing Diabetes Institute, Beijing Tongren Hospital, Capital Medical University, Beijing, 100730, China

^4^ Department of Endocrinology, Shanghai Gongli Hospital, Shanghai, 200135, China

^5^ Department of Endocrinology and Metabolism, Shanghai Fourth People's Hospital Affiliated to Tongji University School of Medicine, Shanghai, 200081, China.

*** Correspondence:**Corresponding Author
Huai-dong Song, huaidong_s1966@163.com (Huai-dong Song);

Gang Wei, gangwei_2013@163.com (Gang Wei)

(Liu Yang, Ping-hui Tu and Gang Wei should be regarded as joint first authors)

**Keywords: Thyroid disruption; hypothyroidism; thyroid histomorphology; hypothalamus-pituitary-thyroid (HPT) axis**

**Table S1. Sequence of probe primers for WISH.**

| **Gene** | **Primer ID** | **Sequence of the primers (5’-3’)** |
| --- | --- | --- |
| *tg* | *tg*-probe-F  *tg*-probe-R | GTACCACTTACCTGAAAACG  TGCTTGGAGTCAGAGTGAAG |
| *tsh* | *tsh*-probe-F  *tsh-*probe-R | TTAATGAAGGTTGCCGTGCC  TCCTCGGGGTACAGATGATG |

**Table S2. Primers and sources of genes related to the HPT axis for qRT-PCR.**

| **Gene symbol** | **Sequence of the primers (5’-3’)** | **Gene Bank number** | **Efficiency (%)** | |
| --- | --- | --- | --- | --- |
| *trh* | Forward: TGGAGCCGGAGGTGAAGA  Reverse: GCAGTGGGGTCCTCTAGCAT | NM_001012365.2 | 104.2 |  |
| *tshβ* | Forward: AGGTTGCCGTGCCTATGTG  Reverse: GACCCACCAACTCCTTTATGT | NM_181494.2 | 100.1 |  |
| *tshr* | Forward: GCGCCAACCCTTTTCTGTAT  Reverse: CTCGTTTGCTCCTGTTTGCT | NM_001145763.2 | 97.8 |  |
| *pax8* | Forward: GAAGATCGCGGAGTACAAGC  Reverse: CTGCACTTTAGTGCGGATGA | AF072549 | 102.4 |  |
| *nis* | Forward: TGGTTGGTGTGGTGGTCAGTTA  Reverse: GCATCGCAGGGCTTTTGTT | NM_001089391.1 | 99.6 |  |
| *tpo* | Forward: ACACGCGTGCAAAAACTCTC  Reverse: CAAAGCTGAGCCTCCTGGAA | NM_001329865.1 | 97.4 |  |
| *tg* | Forward: GCAGAGCCAAGAACATCAAGAAT  Reverse: GGCGAGTGCTGTAAAGAGTAGAAC | DQ278875.1 | 103.3 |  |
| *dio1* | Forward: GGTGGTGGATGAGATGAACAAC  Reverse: TCCGATGCCTCCCTGATAGA | NM_001007283.1 | 96.6 |  |
| *dio2* | Forward: ATTTCTCCTTGCCTCCTCAGTG  Reverse: GCCACCTCCGAACATCTTTAAG | NM_212789.3 | 96.4 |  |
| *trα* | Forward: GGCTCGGAGTGGTTTCTGA  Reverse: CTTGCGGTGGTTGATGTAGTG | NM_131396.1 | 97.2 |  |
| *trβ* | Forward: AGCGTTGTCAGGAGGAGTTTC  Reverse: GATTGGATTGCCATCAGTCTTC | NM_131340.1 | 97.3 |  |
| *ttr* | Forward: CCCAGAGTCGTGGCGAAG  Reverse: CGGGTGGAGTTTGACACTTT | BC081488 | 104.2 |  |
| *ugt1ab* | Forward: CCACCAAGTCTTTCCGTGTT  Reverse: GCAGTCCTTCACAGGCTTTC | NM_213422 | 98.4 |  |
| *gapdh* | Forward: CGCTGGCATCTCCCTCAA  Reverse: TCAGCAACACGATGGCTGTAG | AF057040.1 | 99.3 |  |
